# Production and Characterization of a Pullulan-Based Facial Mask Incorporating Grape Seed Flour Extract for Cosmeceutical Applications

**DOI:** 10.3390/ijms262411845

**Published:** 2025-12-08

**Authors:** Ester Ferreira, Bárbara Encarnação, José Francisco Cascalheira, Eugenia Gallardo, Susana Ferreira, Ana Ramos, Fernanda Domingues, Ângelo Luís

**Affiliations:** 1 RISE-Health, Department of Medical Sciences, Faculty of Health Sciences, University of Beira Interior, Av. Infante D. Henrique, 6200-506 Covilhã, Portugal; ester.ferreira@ubi.pt (E.F.); barbara.encarnacao@ubi.pt (B.E.); egallardo@fcsaude.ubi.pt (E.G.); susana.ferreira@fcsaude.ubi.pt (S.F.); 2 Fiber Materials and Environmental Technologies (FibEnTech-UBI), University of Beira Interior, R. Marquês D’Ávila e Bolama, 6201-001 Covilhã, Portugal; ammr@ubi.pt; 3 RISE-Health, Department of Chemistry, Faculty of Sciences, University of Beira Interior, R. Marquês D’Ávila e Bolama, 6201-001 Covilhã, Portugal; jfcascalheira@ubi.pt (J.F.C.); fdomingues@ubi.pt (F.D.); 4 Pharmaco-Toxicology Laboratory, UBIMedical, University of Beira Interior, Estrada Municipal 506, 6200-284 Covilhã, Portugal; 5 Department of Chemistry, Faculty of Sciences, University of Beira Interior, R. Marquês D’Ávila e Bolama, 6201-001 Covilhã, Portugal

**Keywords:** pullulan, grape seed, facial mask, antioxidant activity, antimicrobial activity, tyrosinase, elastase, resveratrol

## Abstract

This study aimed to develop and characterize an eco-friendly facial mask based on the pullulan biopolymer incorporating grape seed flour extract, a sustainable source of polyphenols. The extract was characterized by its phenolic content, antioxidant capacity, enzyme inhibition and antimicrobial activity. High total phenolic and flavonoid contents, along with the presence of *trans*-resveratrol, conferred strong antioxidant activity. The extract effectively inhibited tyrosinase and elastase enzymes, indicating its anti-aging potential, and exhibited antimicrobial effects particularly against *Staphylococcus aureus*. The incorporation of the extract in pullulan films increased thickness and coloration while maintaining transparency and improving barrier properties. The bioactive films showed strong antioxidant activity and displayed selective antibacterial activity against *S. aureus*, including MRSA strains. A facial mask prototype was successfully produced, demonstrating flexibility, solubility, and potential for topical applications. Overall, the developed pullulan–grape seed extract films exhibit multifunctional cosmeceutical potential combining antioxidant, antimicrobial, and anti-aging effects with sustainable valorization of wine industry by-products.

## 1. Introduction

The skin, the body’s largest organ, acts as a protective barrier against external threats and plays a major role in personal well-being [1]. Among cosmetic products designed to support skin health, facial masks have gained popularity due to their practicality and ability to deliver bioactive compounds directly to the skin surface [1].

Driven by consumer demand for safer and more sustainable formulations, the cosmetic industry has increasingly turned to plant-derived ingredients, which are valued for their antioxidant and anti-inflammatory properties and long-standing use in traditional applications [2,3]. Polyphenols represent one of the most relevant groups of natural bioactive molecules, owing to their strong capacity to neutralize oxidative stress and protect the skin from environmental damage [4].

Grape by-products are an abundant and low-cost source of polyphenols, containing phenolic acids, flavonoids, anthocyanins, procyanidins and flavanols [5,6]. Seeds and skins are especially rich in these compounds, with extractable phenols reaching up to 60–70% of seed dry extract and 28–35% of skin dry extract [5,6]. Such bioactives exhibit antioxidant and antimicrobial properties that make grape-derived extracts attractive for cosmetic formulations [5]. Resveratrol, a key grape polyphenol, is widely studied for its antioxidant, anti-inflammatory and wound-healing activity, and is considered a promising compound for preventing photoaging and promoting skin regeneration [7,8,9,10].

Despite growing interest in the cosmetic application of grape-derived polyphenols, there is still limited knowledge on their incorporation into biopolymeric facial mask systems, particularly those based on pullulan, a biodegradable and transparent polysaccharide suitable for topical delivery. In addition, few studies have evaluated how such natural extracts affect the physicochemical, mechanical, antioxidant, and antimicrobial properties of pullulan films, or their potential contribution to targeting skin-aging–related enzymes.

Therefore, this study aimed to develop and characterize a pullulan-based facial mask incorporating grape seed flour extract as a natural source of polyphenols. The objective was to evaluate the physicochemical, mechanical, antioxidant, and antimicrobial properties of the biopolymeric matrix along with its ability to inhibit enzymes involved in skin aging, exploring the material’s potential as an eco-friendly and effective cosmeceutical product for skin care applications.

## 2. Results and Discussion

### 2.1. Extract Characterization

The extraction of bioactive compounds from plant sources is the initial stage in harnessing phytochemicals for the development of dietary supplements or nutraceuticals, food additives, as well as pharmaceutical and cosmetic formulations [11].

The grape seed flour extract exhibited a relatively high extraction yield (28.80 ± 0.80%), suggesting that the extraction method employed was efficient for recovering phenolic constituents (Table 1). Similar extraction yields were reported in the literature using analogous extraction methods [11].

The total phenolic content (430.00 ± 90.00 mg GAE/g extract) confirms that grape seed flour is an abundant source of phenolic compounds, key contributors to antioxidant capacity (Table 2). The flavonoid content (11.87 ± 1.71 mg QE/g extract) was comparatively lower, indicating that other phenolic classes, such as phenolic acids, stilbenes and tannins, may account for most of the antioxidant activity (Table 2). The presence of *trans*-resveratrol (5.75 ± 0.36 µg/g extract) is noteworthy, as this polyphenol is well recognized for its cardioprotective and anti-aging properties, reinforcing the potential nutraceutical relevance of the extract (Table 2).

In terms of antioxidant performance, the extract exhibited potent radical scavenging activity in the DPPH assay, with an IC_50_ of 23.20 ± 8.51 mg/L and an Antioxidant Activity Index (AAI) of 2.21 ± 0.08, which classifies it as exhibiting *very strong* antioxidant activity (Table 2). Although gallic acid, the reference compound, showed a markedly lower IC_50_ (2.23 ± 0.02 mg/L) and higher AAI (22.77 ± 0.25), the extract’s performance remains remarkable considering its complex phytochemical composition and the likely interactions among phenolic constituents (Table 2). Conversely, in the β-carotene/linoleic acid assay, the extract displayed a markedly higher IC_50_ (935.27 ± 22.53 mg/L) compared to the reference compound BHT (76.95 ± 6.17 mg/L), indicating a less pronounced capacity to inhibit lipid peroxidation (Table 2).

Tyrosinase is a copper-containing metalloenzyme that is expressed across a vast range of species ranging from bacteria and fungi to mammals. It is involved in two sequential reactions of the melanin synthesis pathway. The first reaction is the hydroxylation of a monophenol, and the second reaction is the conversion of an ortho-diphenol to a quinone. Quinone subsequently undergoes a series of reactions, including polymerization, to form melanin in the skin [12]. Development and screening of tyrosinase inhibitors, therefore, is very useful for conditions such as hyperpigmentation and melasma [13]. Skin inflammation involves elastase release by neutrophils and fibroblasts. When the extracellular elastase concentration exceeds the buffering capacity of endogenous inhibitors such as serpins, it causes degradation of a wide range of extracellular matrix proteins, including fibronectin, laminin, proteoglycans, collagens, and elastin, contributing to skin deterioration [13]. Enzyme inhibition assays demonstrated the multifunctional bioactivity of the extract. The grape seed flour extract showed considerable inhibitory activity against tyrosinase (IC_50_ = 168.82 ± 6.57 mg/L) and strong elastase inhibition (IC_50_ = 6.34 ± 0.87 mg/L), highlighting its relevance for cosmetic and dermatological applications targeting hyperpigmentation and skin aging (Table 2).

Overall, the chemical profile and biological properties of the grape seed flour extract underscore its value as a sustainable natural source of bioactive compounds with antioxidant, and skin anti-aging potential. The combination of phenolic richness, significant radical scavenging activity, and enzyme inhibition capacity suggests that this by-product could be effectively used in nutraceutical and cosmeceutical formulations, aligning with sustainable valorization strategies for agro-industrial residues.

The antimicrobial activity of the grape seed flour extract was evaluated against a representative panel of Gram-positive and Gram-negative bacteria, as well as yeast strains (Table 3). The extract exhibited broad-spectrum antimicrobial effects, though with varying intensity across the tested microorganisms, in agreement with previous findings [5,14]. In general, higher susceptibility was observed among Gram-positive bacteria, particularly *Staphylococcus aureus*, whereas Gram-negative strains tended to be more resistant.

Among the Gram-positive bacteria, *S. aureus* ATCC 25923 exhibited inhibition zones of 16.99 ± 0.83 mm. The MIC values for *S. aureus* clinical isolates (SA 01/10, SA 02/10, SA 08) ranged from 2.5 to 10 mg/mL in line with the observed against methicillin-resistant *S. aureus* (MRSA 10/08 and MRSA 12/08) (Table 3). These findings highlight the potential of grape seed flour extract as a natural antimicrobial agent capable of counteracting multidrug-resistant pathogens, albeit at higher concentrations than conventional antibiotics.

The extract was less active against Gram-negative strains, with inhibition zones generally below 12 mm and MIC values above 10 mg/mL for *E. coli* (Table 3). This lesser efficacy is likely attributable to the structural characteristics of Gram-negative bacteria, namely the outer membrane rich in lipopolysaccharides, which acts as a barrier to phenolic compounds [15]. Nevertheless, moderate activity was observed against *A. baumannii* (12.14 ± 1.70 mm; MIC = 5 mg/mL), a clinically relevant opportunistic pathogen often associated with hospital-acquired infections.

The antifungal assays revealed selective inhibition against *Candida* species. Generally, the antimicrobial activity of the extract was better for yeasts than for bacteria considering the obtained MIC (Table 3). These results indicate that the extract contains bioactive molecules capable of compromising fungal cell wall integrity and/or interfering with key metabolic processes, although further phytochemical fractionation would be required to identify the specific compounds responsible.

The extract also exhibited anti-quorum sensing activity, evidenced by an inhibition halo of 1.85 ± 0.35 mm (Table 3), suggests that the grape seed flour extract may modulate bacterial communication systems, potentially reducing virulence factor expression without exerting strong bactericidal effects [16]. Such interference with quorum sensing is particularly relevant in the context of antimicrobial resistance, as it represents a mechanism for attenuating pathogenicity without promoting selective pressure [17].

Overall, the antimicrobial and anti-quorum sensing results complement the strong antioxidant profile previously observed for this extract. The combined biological activities support the potential use of grape seed flour as a multifunctional ingredient in nutraceutical, food preservation, and topical formulations aimed at controlling microbial growth and oxidative stress.

### 2.2. Films’ Characterization

**Figure 1:**
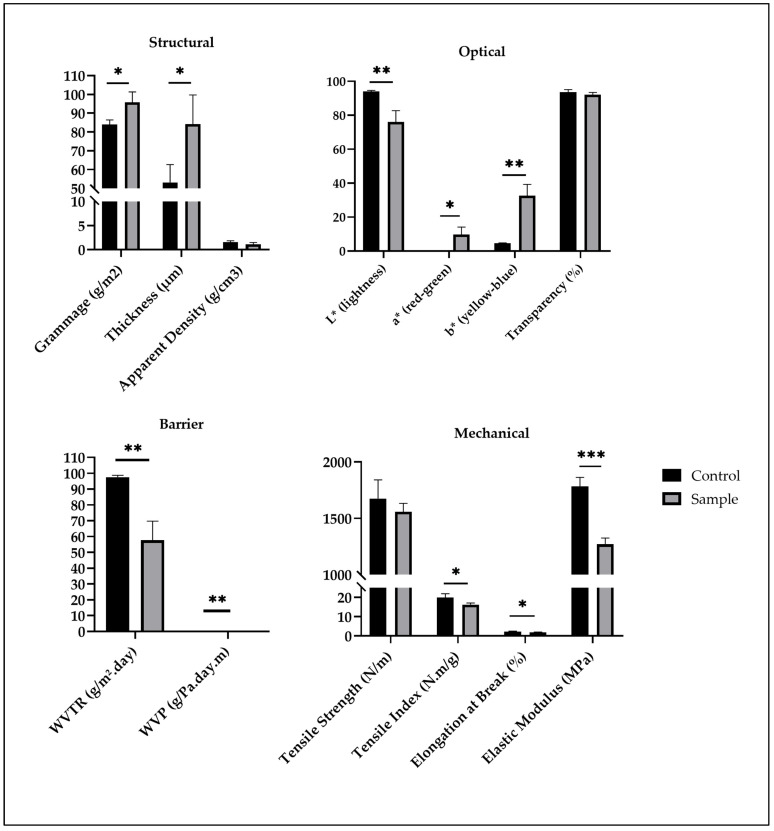
Structural, optical, barrier and mechanical properties of the films (results expressed as mean ± standard deviation). WVP—water vapor permeability; WVTR—water vapor transmission rate; * *p*-value < 0.05; ** *p*-value < 0.01; *** *p*-value < 0.001.

The incorporation of grape seed flour extract significantly affected the physical, mechanical, optical, and functional properties of the films (Figure 1). The grammage and thickness of the films increased from 83.95 ± 2.43 g/m^2^ and 53.13 ± 9.46 µm in the control to 95.76 ± 5.53 g/m^2^ and 84.14 ± 15.55 µm, respectively, in the films containing the extract. This increase is likely associated with the presence of polyphenolic compounds and other macromolecules in the extract, which have altered the polymeric matrix by enhancing intermolecular interactions and reducing chain compactness [18]. Consequently, the apparent density decreased (from 1.58 ± 0.26 to 1.13 ± 0.36 g/cm^3^), suggesting the formation of a less compact microstructure with higher porosity (Figure 1).

The color parameters confirmed the strong pigmentation effect of the extract (Figure 1). The lightness (L*) decreased from 94.05 ± 0.60 to 76.11 ± 6.62, while both a* and b* values increased markedly, indicating a clear shift toward reddish and yellowish tones. Such color modification is consistent with the presence of anthocyanins, flavonoids, and tannins in grape seed extracts, as previously reported for similar plant-derived additives [19]. Despite this chromatic alteration, transparency was only slightly affected, decreasing from 93.66 ± 1.45% to 92.15 ± 1.31%, maintaining high optical clarity desirable for cosmetic applications (Figure 1).

Mechanically, the incorporation of the extract slightly reduced tensile strength (from 1675.67 ± 165.19 to 1557.67 ± 74.55 N/m), tensile index (from 19.95 ± 1.97 to 16.28 ± 0.79 N.m/g), elongation at break (from 2.31 ± 0.18% to 1.92 ± 0.13%), and elastic modulus (from 1782.33 ± 80.21 to 1270.04 ± 55.03 MPa) (Figure 1). These decreases are likely associated with the interference of phenolic compounds in the hydrogen bonding of the film-forming matrix, leading to decreased structural cohesion [19]. Similar mechanical trends have been observed in bio-based films containing polyphenol-rich extracts, where increased heterogeneity and reduced chain limit compromise mechanical reinforcement [5,14].

Conversely, barrier performance improved considerably. The WVTR decreased from 97.50 ± 1.20 to 57.59 ± 12.10 g/m^2^.day, and WVP also slightly decreased from 4.59 × 10^−6^ ± 5.63 × 10^−8^ to 4.28 × 10^−6^ ± 8.99 × 10^−7^ g/Pa.day.m (Figure 1). This enhancement can be attributed to the hydrophobic nature of certain polyphenolic compounds, which likely increased the tortuosity of the diffusion pathway for water molecules through the film matrix [20].

The control pullulan films exhibited thickness, mechanical and barrier properties within the expected range for neat pullulan films reported in the literature [21]. Typically, pullulan films show thicknesses of approximately 40–100 µm, tensile strengths between 30 and 100 MPa, elongation at break from 10 to 70%, and water vapor permeability on the order of 10^−10^ g.m/m^2^.s.Pa. The values obtained in this study fall within these characteristic ranges, confirming that the control formulation behaved as a representative pullulan film and is therefore suitable as a baseline for evaluating the effect of the incorporated grape seed flour extract.

As expected, films containing the extract exhibited remarkable bioactive potential. Total phenolic content reached 1641.27 ± 146.19 mg GAE/m^2^, while total flavonoids were 192.17 ± 8.48 mg QE/m^2^ (Table 4). Moreover, the films contained 72.09 ± 0.49 µg/m^2^ of *trans*-resveratrol, confirming the efficient incorporation of antioxidant compounds from grape seed flour. The antioxidant assays showed high inhibition percentages in both DPPH (85.13 ± 3.88%) and β-carotene/linoleic acid (98.24 ± 0.81%) systems, demonstrating strong radical scavenging and lipid peroxidation inhibitory capacity (Table 4). Overall, the incorporation of grape seed flour extract imparted multifunctional properties to the films, combining antioxidant activity with enhanced barrier performance, thereby reinforcing their potential for cosmetic and biomedical applications.

**Figure 2:**
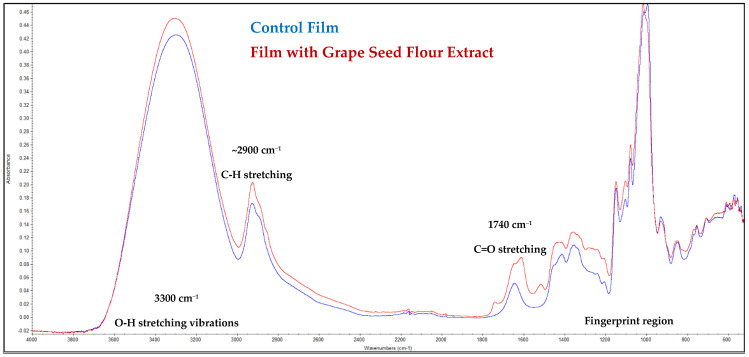
FTIR spectra of the films.

The FTIR spectra of the control film and the film incorporated with grape seed flour extract are presented in Figure 2. Both spectra exhibited similar overall profiles, indicating that the addition of the extract did not substantially modify the polymeric network structure of the film matrix. Nevertheless, subtle differences in the intensity and position of certain absorption bands suggest molecular interactions between the phenolic constituents of the extract and the functional groups of the polymer.

A broad absorption band centered around 3300 cm^−1^ corresponds to O–H stretching vibrations, typically associated with hydroxyl groups from polysaccharides and polyphenols [22]. The slight increase in intensity observed for the extract-containing film suggests the contribution of phenolic hydroxyl groups from the grape seed flour extract, indicating the formation of hydrogen bonds between the film matrix and bioactive compounds [23].

The absorption bands near 2920 cm^−1^ and 2850 cm^−1^, assigned to C–H stretching of aliphatic chains, displayed minor changes in intensity, implying partial interactions or conformational rearrangements in the polymeric backbone. The region around 1740 cm^−1^, attributed to C=O stretching of ester or carboxylic groups, showed a slight shift and intensity variation, which may reflect hydrogen bonding between the extract constituents (such as gallic acid and flavonoids) and the matrix carbonyl groups [22].

In the fingerprint region (1500–800 cm^−1^), characteristic peaks associated with C–O–C and C–O stretching vibrations were observed. The increased absorption in this range for the film containing the extract suggests incorporation of phenolic compounds rich in aromatic and ether linkages [24]. Furthermore, the small differences in the peaks around 1030–1050 cm^−1^ further support the presence of polyphenolic structures and possible cross-linking effects within the polymer network.

Generally, the FTIR results confirm the successful incorporation of grape seed flour extract into the film without disrupting its structural integrity. The observed spectral modifications—particularly in the hydroxyl and carbonyl regions—indicate molecular interactions that could contribute to enhanced stability, antioxidant capacity, and potential antimicrobial performance of the functionalized film.

**Figure 3:**
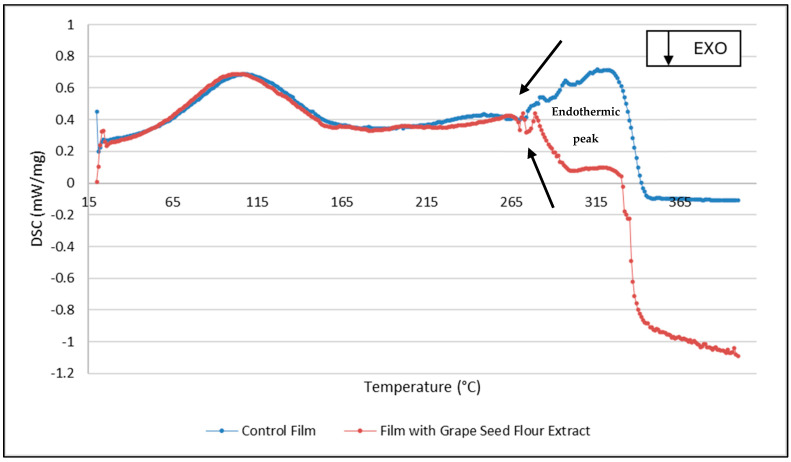
DSC thermograms of the films.

The DSC thermograms revealed distinct thermal transitions between control and grape seed flour extract-containing films (Figure 3). The films with the extract showed a broader and slightly shifted endothermic peak, suggesting increased structural heterogeneity and possible interactions between phenolic compounds and the film matrix [23]. The decrease in peak intensity and the shift to lower temperatures indicate partial plasticization and reduced crystallinity, likely caused by hydrogen bonding between extract molecules and polymer chains. This thermal behavior corroborates the mechanical data, confirming a more amorphous and flexible structure, consistent with the observed reductions in elastic modulus and tensile strength.

The antimicrobial activity of the control film and the film incorporated with grape seed flour extract was evaluated against a panel of Gram-positive and Gram-negative bacteria, as well as yeast strains (Table 5). The control film did not exhibit any inhibitory effect. Conversely, the extract-containing film displayed a selective inhibitory effect, particularly against *Staphylococcus aureus* strains (Table 5).

Among the tested microorganisms, *S. aureus* ATCC 25923 showed inhibition zones of 10.23 ± 0.01 mm. The effect was also noticeable in clinical isolates of *S. aureus*, with inhibition zones ranging from 8.41 ± 0.52 mm (*S. aureus* SA 02/10) to 12.73 ± 0.59 mm (*S. aureus* SA 08). Notably, both methicillin-sensitive and methicillin-resistant *S. aureus* (MRSA 10/08 and MRSA 12/08) were also inhibited by the film incorporated with grape seed flour extract (Table 5), suggesting that the active compounds present in the extract can act independently of the resistance mechanisms.

In contrast, no inhibitory effect was observed against *E. coli*, *P. aeruginosa*, *A. baumannii*, or *Candida* spp., as the inhibition zones remained at 6.00 ± 0.00 mm (Table 5). This selective antimicrobial spectrum indicates that the phenolic constituents in the grape seed extract—such as proanthocyanidins, catechins, and gallic acid derivatives—are more effective against Gram-positive bacteria. This phenomenon has been associated with the presence of a thicker peptidoglycan layer in Gram-positive cell walls, which facilitates the interaction of phenolic compounds with bacterial membranes, leading to structural damage and cell death [25]. Conversely, the outer lipopolysaccharide layer of Gram-negative bacteria acts as a permeability barrier, restricting the diffusion of these bioactive molecules [26].

The anti-quorum sensing assay showed no detectable activity (0.00 ± 0.00), suggesting that the observed antimicrobial effect of the extract is primarily bactericidal or bacteriostatic rather than quorum-sensing inhibition (Table 5).

The antimicrobial properties of the grape seed flour extract-based films suggest promising applications in dermatological and wound-care formulations. The selective activity against *S. aureus*, including MRSA strains, is of particular clinical relevance, as *S. aureus* is a common cause of skin and soft tissue infections and is frequently isolated from chronic wounds, burns, and atopic dermatitis lesions [27].

Polyphenols from grape seed extracts are known for their anti-inflammatory, antioxidant, and collagen-stabilizing activities, which contribute to enhanced wound healing and protection against oxidative stress [28]. Their dual antioxidant–antimicrobial functionality could help prevent infection while minimizing oxidative damage in wound microenvironments. When incorporated into a biopolymeric film, these compounds can provide a controlled and sustained release, maintaining local antimicrobial action while supporting tissue regeneration.

### 2.3. Facial Mask Prototype

**Figure 4:**
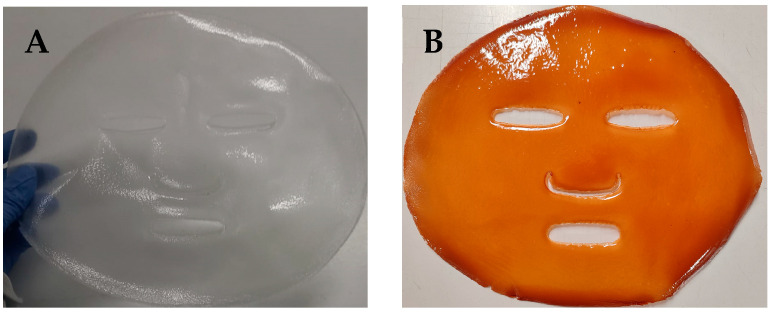
Photographs of the facial mask prototypes produced: (**A**)—control film; (**B**)—film with grape seed flour extract.

In this study, facial mask prototypes using the developed films incorporating grape seed flour extract were successfully produced (Figure 4). As shown in the photographs, masks feature openings for the eyes, nose, and mouth, allowing them to easily conform to the contours of the human face, since they present pseudoplastic properties for a handy application (Figure 4). For use, the face should be lightly moistened with water before applying the mask directly onto the skin. After approximately 30 min, the face can be rinsed with water, as the pullulan-based film readily dissolves upon contact with moisture, leaving no residue. This feature enhances the product’s practicality and user compliance, while the incorporated grape seed extract contributes potential antioxidant and photoprotective benefits during application.

Pullulan is an edible microbial polymer approved for use in food products by regulatory authorities worldwide. Beyond the food industry, pullulan and its derivatives have potential in drug and gene delivery, wound healing, and tissue engineering [19]. This polymer is non-ionic, non-hygroscopic, non-toxic, and exhibits no mutagenic or carcinogenic effects. These properties render pullulan a versatile material for applications in food, pharmaceutical, cosmetic and biomedical fields [19].

The developed facial masks are expected to provide multiple skin benefits, as the bioactive properties of the grape seed flour extract were preserved upon incorporation into the pullulan-based films. These bioactivities, including photoprotection, antioxidant, antimicrobial and enzymes-inhibiting activity, suggest that the masks could help protect the skin from oxidative stress, improve hydration, and promote overall skin health. Moreover, the pullulan matrix ensures efficient delivery of the extract to the skin, enhancing the potential efficacy of the masks during use.

Water solubility reflects the resistance of the films to water exposure. The produced facial masks prototypes showed a water solubility of near 100%, indicating the stronger affinity to water of these films.

Although pullulan is widely recognized as a biocompatible and non-toxic polysaccharide, and the incorporated bioactive compounds have established safety profiles, a complete assessment of skin behavior requires dedicated in vitro assays. Relevant evaluations include keratinocyte and fibroblast cytotoxicity, irritation potential using reconstructed human epidermis models, and genotoxicity tests. These analyses were beyond the scope of the present physicochemical characterization but represent an important next step to confirm the safety of the films for topical applications.

## 3. Materials and Methods

### 3.1. Grape Seed Flour Extract

The grape seed flour was obtained from Raab Vitalfood GmbH (Rohrbach, Germany). It was produced by milling defatted grape seed obtained through careful cold pressing of sun-ripened grape seeds. To prepare the extract, 5 g of grape seed flour were placed in an Erlenmeyer flask with 100 mL of ethanol 80% (v,v). The mixture was subjected to sonication in an ultrasonic bath for 1 h at 30 °C. Afterwards, the mixture was vacuum filtered using a D2 porous plate filter, and the solvent was evaporated under reduced pressure using a rotary evaporator at 45 °C and 150 mbar. The resulting dried extract was stored in a desiccator until further use.

### 3.2. Determination of Total Phenolic Compounds

The total phenolic content of the extract was quantified using the Folin–Ciocalteu colorimetric assay. In this procedure, 50 μL of the extract (50 mg/mL in methanol) (or gallic acid standard solutions) were mixed with 450 μL of distilled water. Subsequently, 2.5 mL of 0.2 N Folin–Ciocalteu reagent were added, and the mixture was allowed to react for 5 min. Then, 2 mL of aqueous sodium carbonate solution (75 g/L) were incorporated. The resulting reaction mixtures were incubated for 90 min at 30 °C, after which the absorbance was measured at 765 nm using a UV–Vis spectrophotometer. A standard calibration curve was constructed with gallic acid solutions prepared in methanol. The total phenolic content was expressed as milligrams of gallic acid equivalents per gram of extract (mg GAE/g extract). All measurements were performed in triplicate [29].

### 3.3. Determination of Flavonoids Content

The aluminum chloride colorimetric assay was employed to quantify the flavonoid content as previously described [14]. In this method, 500 μL of the extract (50 mg/mL in methanol) were mixed with 1.5 mL of methanol, 0.1 mL of 10% (w,v) aluminum chloride solution, 0.1 mL of 1 M potassium acetate, and 2.8 mL of distilled water. The reaction mixtures were incubated at room temperature for 30 min, after which the absorbance was recorded at 415 nm using a UV–Vis spectrophotometer. A standard calibration curve was generated using quercetin solutions prepared in methanol. The results were expressed as milligrams of quercetin equivalents per gram of extract (mg QE/g extract). All measurements were performed in triplicate [29].

### 3.4. Quantification of trans-Resveratrol by HPLC-DAD

The identification and quantification of *trans*-resveratrol present in the grape seed flour extract were carried out by High-Performance Liquid Chromatography with Diode-Array Detection (HPLC-DAD) as previously described [15]. Chromatographic analysis was performed using an Agilent Technologies 1290 HPLC system (Soquimica, Lisbon, Portugal) equipped with a diode array detector (DAD) (Soquimica, Lisbon, Portugal). Separation was achieved on a YMC-Triart PFP reversed-phase column (150 × 4.6 mm i.d., 5 µm, 12 nm) fitted with a Triart PFP guard column (10 × 3.0 mm i.d., 5 µm, 12 nm), both supplied by Solitica (Lisbon, Portugal). The mobile phase consisted of acetonitrile (line A) and 0.1% trifluoroacetic acid (line B), using the following gradient: 10% A for 15 min; 15% A at 15–20 min; 18% A at 25 min; 30% A at 45 min; 42% A at 50 min; 50% A at 54 min; 100% A at 55 min; and re-equilibration at 10% A until 60 min. The flow rate was set at 1.0 mL/min, the column temperature was maintained at 40 °C, and the injection volume was 50 µL. The identification of *trans*-resveratrol was confirmed at a retention time of 38 min and a wavelength of 291 nm, ensuring unequivocal detection under the optimized chromatographic conditions [30]. A calibration curve with several solutions of *trans*-resveratrol was initially obtained.

### 3.5. Antioxidant Activity Evaluation

#### 3.5.1. DPPH Free Radical Scavenging Assay

The antioxidant activity of the extract and gallic acid (reference compound) was evaluated using the 2,2-diphenyl-1-picrylhydrazyl (DPPH) radical scavenging assay, following the procedure described previously with slight modifications [31].

Briefly, 0.1 mL of extract in methanol or standard solution was mixed with 3.9 mL of DPPH methanolic solution. The mixtures were incubated for 90 min at room temperature, and the absorbance was measured at 517 nm using a UV–Vis spectrophotometer. The radical scavenging activity (I%) was calculated according to the following equation:

(1) I(%) =× 100

where A_0_ is the absorbance of the control and A_1_ is the absorbance of the sample at different concentrations. The IC_50_ was determined graphically from the linear portion of the inhibition curve (scavenging percentage vs. extract concentration).

The Antioxidant Activity Index (AAI) was calculated as:

(2) $$AAI=\frac{final\;\mathrm{concentration}\;\mathrm{of}\;\mathrm{DPPH}\;\mathrm{in}\;\mathrm{control}}{IC_{50}}$$

Antioxidant activity was classified as follows: AAI ≤ 0.5 (poor), 0.5–1.0 (moderate), 1.0–2.0 (strong), and AAI ≥ 2.0 (very strong) [29,31]. All assays were performed in triplicate.

#### 3.5.2. β-Carotene Bleaching Assay

The antioxidant potential of the extract was further evaluated using the β-carotene bleaching assay, following a previously described method [29].

Twenty μL a of β-carotene solution (50 mg/mL in chloroform) were mixed with 40 μL of linoleic acid, 400 mg of Tween 40, and 1 mL of chloroform. The mixture was evaporated at 45 °C for 5 min, and the residue was diluted with 100 mL of distilled water saturated with oxygen. Five mL of the emulsion were mixed with 300 μL of extract solutions and butylated hydroxytoluene (BHT) used as the reference standard. The mixtures were incubated at 50 °C for 2 h. Absorbances were measured at 470 nm at t = 0 h and t = 2 h. The antioxidant activity was expressed as the percentage of inhibition of β-carotene oxidation, calculated according to the following equation:

(3) %Inhibition = ×100

where $A_{\mathrm{sample},t\;=\;2}$ and $A_{\mathrm{control},t\;=\;2}$ are the absorbances of the sample and control at the end of incubation, respectively, and $A_{\mathrm{control},t\;=\;0}$ is the initial absorbance of the control at time zero [29].

### 3.6. Evaluation of Antimicrobial and Anti-Quorum Sensing Activities

The antimicrobial activity was evaluated against 12 microbial strains (Gram-positive: *Staphylococcus aureus* ATCC 25923; Clinical isolates of *S. aureus*: SA 01/10, SA 02/10, SA 03/10 and SA 08; Clinical methicillin-resistant *S. aureus*: MRSA 10/08 and MRSA 12/08; Gram-negative: *Escherichia coli* ATCC 25922, *Pseudomonas aeruginosa* ATCC 27853, *Acinetobacter baumannii* LMG 1025; and two yeast strains: *Candida albicans* ATCC 90028 and *Candida tropicalis* ATCC 750). Stock cultures were maintained in 20% glycerol and stored at −80 °C. Prior to antimicrobial testing, all strains were subcultured on the appropriate agar medium. Brain Heart Infusion Agar (BHI) was used for bacterial growth, while Sabouraud Dextrose Agar (SDA) was employed for yeast cultivation.

#### 3.6.1. Disk Diffusion Assay

The antimicrobial activity of the extract was assessed using the disk diffusion method following the CLSI M2-A8 protocol for bacteria and the CLSI M44-A2 for yeasts. To prepare the inoculum, bacterial or fungal cells were suspended in saline to achieve a turbidity equivalent to 0.5 McFarland standard (approximately 1–2 × 10^8^ CFU/mL for non-fastidious bacteria and 1–5 × 10^6^ CFU/mL for yeasts). Sterile disks (6 mm in diameter) were loaded with 20 µL of the extract solution (4 mg extract/disk) and then placed on the agar plates inoculated with the test microorganisms. Dimethyl sulfoxide (DMSO), used as the extract solvent, served as the negative control, while tetracycline (30 µg/disk) and amphotericin B (25 µg/disk) were used as positive controls for bacteria and yeasts, respectively. The bacterial plates were incubated at 37 °C for 24 h, and yeast plates for 48 h under the same temperature conditions. Following incubation, inhibition zones were observed and measured using a digital caliper. All assays were conducted in triplicate [29].

#### 3.6.2. Resazurin Microtiter Method

The antimicrobial potential of the extract was evaluated using the resazurin microtiter assay. For bacterial assays, 100 µL of the extract (dissolved in 10% *v*/*v* DMSO at a stock concentration of 20 mg/mL in Müeller–Hinton Broth, MHB) was added to the first row of wells, ensuring that the final DMSO concentration did not exceed 5%. All remaining wells received 50 µL of MHB. Serial twofold dilutions were then prepared, resulting in wells containing 50 µL of the extract in decreasing concentrations. To each well, 10 µL of resazurin indicator solution (0.1%, *w*/*v* in MHB) and 30 µL of fresh MHB were added, followed by 10 µL of a bacterial suspension adjusted to 0.5 McFarland. All assays were carried out in triplicate and incubated at 37 °C for 24 h. For yeast assays, resazurin was similarly used as a growth indicator, with 50 µL of a sterile resazurin solution (20 mg/mL in water) added to the working suspension (inoculum adjusted to 0.5 McFarland and diluted 1:1000 in RPMI-1640 medium). The microdilution test for yeasts followed the same general procedure as for bacteria, except that the final volume per well was 200 µL. Plates were prepared in triplicate and incubated at 37 °C for 24 h. After incubation, color changes from purple to pink or colorless indicates microbial growth. Tetracycline and amphotericin B were used as controls for bacteria and yeast, respectively. The minimum concentration of the extract that prevented color change was recorded as the Minimum Inhibitory Concentration (MIC) [29].

#### 3.6.3. Evaluation of the Anti-Quorum Sensing Activity

The anti-quorum sensing activity of the extract was evaluated using the biomonitor strain *Chromobacterium violaceum* ATCC 12472. A bacterial suspension was prepared by cultivating *C. violaceum* ATCC 12472 overnight under aerobic conditions at 30 °C with shaking at 250 rpm in Luria–Bertani (LB) broth. The resulting culture was adjusted to an optical density of 1.0 at 620 nm (OD_620_), and LB agar plates were inoculated with this suspension. Sterile disks (6 mm in diameter) were impregnated with 20 µL of the extract and placed on the inoculated plates, which were then incubated at 30 °C for 24 h. Disks containing DMSO served as negative controls and with resveratrol (5 µg/disk) as positive control. Following incubation, the inhibition of violacein pigment production was examined, characterized by a colorless zone of viable cells surrounding the disk. All assays were performed in triplicate [32].

### 3.7. Evaluation of Tyrosinase Activity Inhibition

The tyrosinase inhibitory potential of the extract was evaluated using the Tyrosinase Assay Kit (Catalog No. MAK550, Sigma-Aldrich, Merck, Darmstadt, Germany) according to the manufacturers’ instructions [33]. This assay employs a colorless substrate solution that develops a strong absorption at 510 nm upon enzymatic reaction with tyrosinase. The increase in absorbance at 510 nm is directly proportional to the tyrosinase activity. Briefly, 50 μL of each tyrosinase standard, blank (Assay Buffer), and test samples were added to separate wells of a 96-well microplate. Then, 50 μL of Tyrosinase Substrate working solution were added to each well containing a standard, blank, and test sample to make the total assay volume of 100 μL/well. The reaction mixtures were incubated at room temperature for 3 h and the increase in absorbance was measured at 510 nm using a microplate reader (Bio-Rad xMark, Hercules, CA, USA). The absorbance reading from the blank well was used as a negative control, and its value was subtracted from the standards to obtain the base-line corrected values. A standard calibration curve was constructed, and the corresponding regression equation was used to calculate the tyrosinase activity in the test samples [33]. To evaluate the inhibitory capability of the grape seed extract, an enzyme assay was performed where tyrosinase at 120 U/mL activity was incubated in the absence (control) or in the presence of different concentrations (0.03 to 0.24 g/L in water) of the extract. The IC_50_—which corresponds to the extract concentration that produced a 50% reduction in tyrosinase initial activity—was calculated from the Hill plot of 1/activity vs. extract concentration.

### 3.8. Evaluation of Elastase Activity Inhibition

The potential of the extract to inhibit the elastase was evaluated using the Neutrophil Elastase Activity Assay Kit (Fluorometric) (Catalog No. MAK246, Sigma-Aldrich, Merck, Darmstadt, Germany) according to the manufacturers’ instructions [34]. This kit exploits the enzymatic activity of Neutrophil Elastase (NE) to proteolytically cleave a synthetic substrate and release a fluorophore, which can be easily quantified by fluorescence.

Briefly, 50 μL of each NE standard, blank (Assay Buffer), and test samples were added to separate wells of a 96-well microplate. Then, 50 μL of NE Substrate mix solution were added to each well containing standard, blank, and test samples to make the total assay volume of 100 μL/well. The reaction mixtures were incubated at 37 °C for 20 min and the increase in emitted fluorescence at 500 nm (excitation at 380 nm) was measured using a microplate fluorimeter reader (Spectramax Gemini XS spectrofluorometer, Molecular Devices LLC, San Jose, CA, USA). The fluorescence rate increase was calculated from the slope from the fluorescence vs. time plot, after subtracting the fluorescence of the blank. A standard calibration curve was constructed using different NE activities (0.05–0.25 ng/µL final concentration), and the corresponding regression equation was used to calculate the NE activity in the test samples. To evaluate the inhibitory capacity of the grape seed extract, an enzyme assay was performed where NE at 0.25 ng/µL activity was incubated in the absence (control) or in the presence of different concentrations of grape seed extract. The IC_50_ was calculated from the Hill plot of 1/activity vs. extract concentration.

### 3.9. Preparation of Films

The films were prepared by dissolving 3 g of pullulan (CAS Number: 9057-02-7; TCI Europe N.V., Haven, Belgium) in 100 mL of distilled water (3%, *w*/*v*) under magnetic stirring for 5 min at room temperature. Subsequently, 0.45 g of glycerol (15%, *w*/*w* relative to pullulan) was added, and the solution was continuously stirred for another 30 min at 50 °C. Thereafter, 1 g of grape seed flour extract was incorporated into the mixture, which was maintained under the same conditions for 10 min. The resulting film-forming solution was distributed into six polystyrene Petri dishes (≈16 mL per dish) and dried in a ventilated oven at 60 °C for approximately 2.5 h to allow solvent evaporation and film formation. Control films without the incorporation of grape seed flour extract were also prepared. All films were conditioned at 23 ± 2 °C and 50 ± 5% relative humidity (RH).

### 3.10. Films’ Characterization

#### 3.10.1. Grammage, Thickness and Apparent Density

The grammage of the films was determined by calculating the ratio between their weight and surface area (g/m^2^), following the ISO 536:1995 standard [35,36]. Thickness (µm) was measured using a digital micrometer (Adamel Lhomargy Model MI 20, Veenendaal, The Netherlands) following the ISO 534:2011 standard [37], obtaining five random readings for each film [35]. Following the same standard protocol, the apparent density (g/cm^3^) of the films was determined by the ratio between grammage and thickness [35].

#### 3.10.2. Optical, Mechanical and Barrier Properties

The optical properties of the films including color coordinates and transparency were analyzed using a spectrophotometer (Technidyne Color Touch 2, New Albany, OH, USA). Measurements were taken at a minimum of three random points on each film, employing a D65 standard illuminant and a 10° observer angle. The color coordinates L* (lightness), a* (red-green), and b* (yellow-blue) were recorded. Film transparency was determined following the equation specified in the ISO 22891 standard [38,39].

The mechanical properties of the films—including tensile strength (N/m), tensile index (N.m/g), elongation at break (%), and elastic modulus (MPa)—were evaluated using a tensile testing machine (Thwing-Albert Instrument Co., West Berlin, NJ, USA), following ISO 1924/2 guidelines [40]. The test setup was modified by setting the initial grip separation to 50 mm and the crosshead speed to 10 mm/min [41].

The water vapor permeability (WVP; g/Pa.day.m) and water vapor transmission rate (WVTR; g/m^2^.day) were measured following the ASTM E96-22 standard [42]. The films were sealed over the tops of test cups containing 15 g of anhydrous CaCl_2_ previously dried at 105 °C as desiccant. The cups were then placed in a controlled environment chamber maintained at 23 ± 2 °C and 50 ± 5% RH. Weight variations were recorded every 2 h over a 48 h period. The water vapor transmission gradient was determined from the slope of the linear regression of weight gain as a function of time [43]. All measurements were performed in triplicate.

#### 3.10.3. Fourier-Transform Infrared Spectroscopy (FTIR)

The Fourier Transform Infrared (FTIR) spectra of the films were recorded using a Nicolet iS10 spectrometer equipped with a Smart iTR Basic accessory (Thermo Fisher Scientific, Waltham, MA, USA). Spectra of the films were collected in the range of 4000–600 cm^−1^, acquiring 120 scans with 4 cm^−1^ of resolution [44].

#### 3.10.4. Differential Scanning Calorimetry (DSC)

Differential Scanning Calorimetry (DSC) thermograms of the films were recorded using a calorimeter Netzsch DSC 204 (GWP, Munich, Germany) under an inert atmosphere, with a heating rate of 5 °C/min, and a temperature ranging from 20 to 400 °C. Before the study, samples of the films were maintained at 105 °C for 24 h to totally evaporate the water, also obtaining the respective baselines [44].

#### 3.10.5. Determination of the Total Phenolic Compounds and Flavonoids

The determination of total phenolic compounds and flavonoids in pullulan films containing the grape seed flour extract was carried out according to the procedures described previously for the extract, with some modifications. A film sample (0.5 g) was cut into small fragments and was then dissolved in 20 mL of distilled water under magnetic stirring at room temperature until complete solubilization. The resulting solution was used in the Folin–Ciocalteu and aluminum chloride assays.

#### 3.10.6. Quantification of *trans*-Resveratrol by HPLC-DAD

The quantification of *trans*-resveratrol in the films followed the same HPLC-DAD procedure employed for the extract, with the only modification that the sample consisted of an entire film dissolved in 3 mL of ultrapure water.

#### 3.10.7. Evaluation of Antioxidant, Antimicrobial and Anti-Quorum Sensing Activities

The antioxidant activity of the films was evaluated using the same methods described for the extract, substituting the extract solution by 6 disks (6 mm in diameter) of the films [45].

The antimicrobial and anti-quorum sensing activities of the films were evaluated using the disk diffusion assay, in which the film disks (6 mm in diameter) were placed on inoculated Petri dishes [45].

### 3.11. Production of the Facial Mask Prototype

A prototype facial mask was produced using a professional silicone mold (320.45 cm^2^) with an oval shape and appropriately sized openings for the eyes, nose, and mouth. The pullulan-based film containing grape seed flour extract was prepared as described above, and 150 mL of this film-forming solution was cast onto the silicone mold.

#### Water Solubility

The water solubility of the facial mask prototypes was defined by the content of dry matter solubilized after 24 h of immersion in water. The initial dry matter content of each film was determined by drying to constant weight in an oven at 105 °C. Two disks of films (2 cm of diameter) were cut, weighed and immersed in 50 mL of water. After 24 h of immersion at 23 °C with occasional magnetic stirring, the pieces of films were taken out (by filtration) and dried to constant weight in an oven at 105 °C, to determine the weight of dry matter which was not solubilized in water. The measurement of solubility of the films was determined as percentage [46].

### 3.12. Statistical Analysis

Data were expressed as mean ± standard deviation from at least 3 independent experiments. The significance of the differences between the means obtained in two different conditions was evaluated by Student’s *t*-test by using Microsoft Excel for Windows. Regression analysis was performed using the SPSS Statistics for Windows program version 25.

## 4. Conclusions

This work successfully developed a biodegradable pullulan-based facial mask incorporating grape seed flour extract as a natural cosmeceutical ingredient. The extract showed strong antioxidant, enzyme-inhibitory, and moderate antimicrobial properties, mainly against *Staphylococcus aureus* strains, including resistant isolates. When incorporated into pullulan films, it enhanced the barrier, and functional properties of the material without compromising its structural integrity. The resulting films exhibited high phenolic content and strong radical scavenging ability, confirming the preservation of the bioactivity after incorporation. The facial mask prototype demonstrated mechanical flexibility and water solubility, suggesting its suitability for topical use. These findings highlight the potential of grape seed flour—a wine industry by-product—as a sustainable source of multifunctional ingredients for eco-friendly cosmeceutical formulations. Future studies should address in vivo assessments of skin compatibility, antioxidant performance, and controlled release behavior to validate its practical applicability in cosmetic and dermatological products.

Beyond these laboratory-scale achievements, the findings highlight promising real-world development opportunities. Grape seed flour—a readily available by-product of the wine industry—emerges as a sustainable, low-cost source of multifunctional ingredients suitable for eco-friendly cosmeceutical formulations. Its use could support circular-economy strategies while reducing reliance on synthetic additives. To advance toward commercial viability, future work should explore scalability of the extraction and film-manufacturing processes, long-term stability of the formulations, and compliance with cosmetic regulatory frameworks. Market acceptance studies, focusing on consumer perception of biodegradable masks and upcycled ingredients, would further clarify the innovation’s potential. Additionally, assessments of skin compatibility, antioxidant efficacy, and controlled-release behavior remain essential to validate its practical application in cosmetic and dermatological products.

## Figures and Tables

**Table 1:** Chemical profile of grape seed flour extract (results expressed as mean ± standard deviation).

Parameters	Grape Seed Flour Extract
Extraction Yield (%)	28.80 ± 0.80
Total Phenolics (mg GAE/g extract)	430.00 ± 90.00
Flavonoids (mg QE/g extract)	11.87 ± 1.71
*trans*-Resveratrol (µg/g extract)	5.75 ± 0.36

GAE—gallic acid equivalents; QE—quercetin equivalents.

**Table 2:** Biological activities of grape seed flour extract (results expressed as mean ± standard deviation).

Parameters		Grape Seed Flour Extract	Gallic Acid ^a^/BHT ^b^
DPPH	IC_50_ (mg/L)	23.20 ± 8.51	2.23 ± 0.02 ^a^*
	AAI	2.21 ± 0.08	22.77 ± 0.25 ^a^****
	Antioxidant Activity	Very Strong	Very Strong ^a^
β-Carotene/Linoleic Acid	IC_50_ (mg/L)	935.27 ± 22.53	76.95 ± 6.17 ^b^****
Tyrosinase Inhibition	IC_50_ (mg/L)	168.82 ± 6.57	-
Elastase Inhibition	IC_50_ (mg/L)	6.34 ± 0.87	-

AAI—Antioxidant Activity Index; DPPH—2,2-diphenyl-1-picrylhydrazyl; * *p*-value < 0.05; **** *p*-value < 0.0001.

**Table 3:** Antimicrobial and anti-quorum sensing activities of grape seed flour extract (results expressed as mean ± standard deviation; MIC values expressed as modal values).

Strains	Grape Seed Flour Extract		Tetracycline ^a^/Amphotericin B ^b^/ Resveratrol ^c^	
	Diameter of Inhibition Zone (mm)	MIC (mg/mL)	Diameter of Inhibition Zone (mm)	MIC (µg/mL)
*Staphylococcus aureus* ATCC 25923	16.99 ± 0.83	>10	30.25 ± 0.50 ^a^	0.06 ^a^
*S. aureus* SA 01/10	15.11 ± 0.17	2.5	14.33 ± 0.21 ^a^	0.12 ^a^
*S. aureus* SA 02/10	15.57 ± 0.74	2.5	15.34 ± 0.33 ^a^	0.12 ^a^
*S. aureus* SA 03/10	13.27 ± 0.24	>10	17.78 ± 0.40 ^a^	0.12 ^a^
*S. aureus* SA 08	20.92 ± 2.21	2.5	16.37 ± 0.39 ^a^	0.12 ^a^
MRSA 10/08	15.18 ± 0.60	2.5	10.18 ± 0.12 ^a^	0.50 ^a^
MRSA 12/08	14.40 ± 0.35	10	12.26 ± 0.14 ^a^	0.50 ^a^
*Escherichia coli* ATCC 25922	6.59 ± 1.01	>10	23.25 ± 0.50 ^a^	0.06 ^a^
*Pseudomonas aeruginosa* ATCC 27853	9.68 ± 1.74	>10	11.50 ± 0.58 ^a^	0.25 ^a^
*Acinetobacter baumannii* LMG 1025	12.14 ± 1.70	5	25.63 ± 0.25 ^a^	0.06 ^a^
*Candida albicans* ATCC 90028	6.00 ± 0.00	0.08	20.33 ± 0.58 ^b^	0.25 ^b^
*Candida tropicalis* ATCC 750	13.53 ± 1.43	0.156	21.50 ± 0.58 ^b^	0.50 ^b^
Anti-QS	1.85 ± 0.35	-	8.93 ± 0.23 ^c^	-

MIC—minimum inhibitory concentration; MRSA—methicillin-resistant *Staphylococcus aureus*; QS—quorum sensing.

**Table 4:** Bioactive properties of the films (Results expressed as mean ± standard deviation).

Properties	Film with Grape Seed Flour Extract
Total Phenolics (mg GAE/m^2^)	1641.27 ± 146.19
Flavonoids (mg QE/m^2^)	192.17 ± 8.48
*trans*-Resveratrol (µg/m^2^)	72.09 ± 0.49
DPPH (% Inhibition)	85.13 ± 3.88
β-Carotene/Linoleic Acid (% Inhibition)	98.24 ± 0.81

GAE—gallic acid equivalents; QE—quercetin equivalents.

**Table 5:** Antimicrobial and anti-quorum sensing activities of the films (Results expressed as mean ± standard deviation).

Strains	Diameter of Inhibition Zone (mm)	
	Control Film	Film with Grape Seed Flour Extract
*Staphylococcus aureus* ATCC 25923	6.00 ± 0.00	10.23 ± 0.01
*S. aureus* SA 01/10	6.00 ± 0.00	9.11 ± 0.35
*S. aureus* SA 02/10	6.00 ± 0.00	8.41 ± 0.52
*S. aureus* SA 03/10	6.00 ± 0.00	8.45 ± 0.60
*S. aureus* SA 08	6.00 ± 0.00	12.73 ± 0.59
MRSA 10/08	6.00 ± 0.00	9.11 ± 0.26
MRSA 12/08	6.00 ± 0.00	8.42 ± 0.83
*Escherichia coli* ATCC 25922	6.00 ± 0.00	6.00 ± 0.00
*Pseudomonas aeruginosa* ATCC 27853	6.00 ± 0.00	6.00 ± 0.00
*Acinetobacter baumannii* LMG 1025	6.00 ± 0.00	6.00 ± 0.00
*Candida albicans* ATCC 90028	6.00 ± 0.00	6.00 ± 0.00
*Candida tropicalis* ATCC 750	6.00 ± 0.00	6.00 ± 0.00
Anti-QS	0.00 ± 0.00	0.00 ± 0.00

QS—quorum sensing.

## Data Availability

The original contributions presented in this study are included in the article. Further inquiries can be directed to the corresponding author.

## References

[1] Nilforoushzadeh M.A., Amirkhani M.A., Zarrintaj P., Moghaddam A.S., Mehrabi T., Alavi S., Sisakht M.M. (2018). Skin care and rejuvenation by cosmeceutical facial mask. J. Cosmet. Dermatol..

[2] Patil B.S., Jayaprakasha G.K., Chidambara Murthy K.N., Vikram A. (2009). Bioactive compounds: Historical perspectives, opportunities, and challenges. J. Agric. Food Chem..

[3] Michalak M. (2022). Plant-derived antioxidants: Significance in skin health and the ageing process. Int. J. Mol. Sci..

[4] Tomás-Barberán F.A., Andrés-Lacueva C. (2012). Polyphenols and health: Current state and progress. J. Agric. Food Chem..

[5] Ivanov Y., Godjevargova T. (2024). Antimicrobial polymer films with grape seed and skin extracts for food packaging. Microorganisms.

[6] Teixeira A., Baenas N., Dominguez-Perles R., Barros A., Rosa E., Moreno D.A., Garcia-Viguera C. (2014). Natural Bioactive Compounds from Winery By-Products as Health Promoters: A Review. Int. J. Mol. Sci..

[7] Lin M.H., Hung C.F., Sung H.C., Yang S.C., Yu H.P., Fang J.Y. (2021). The bioactivities of resveratrol and its naturally occurring derivatives on skin. J. Food Drug Anal..

[8] Rimando A.M., Kalt W., Magee J.B., Dewey J., Ballington J.R. (2004). Resveratrol, pterostilbene, and piceatannol in *Vaccinium* berries. J. Agric. Food Chem..

[9] Fabbrocini G., Satibano S., Rosa G.D., Battimiello V., Fardella N., Ilardi G., Rotonda M.I.L., Longobardi A., Mazzella M., Siano M. (2011). Resveratrol-containing gel for the treatment of acne vulgaris. Am. J. Clin. Dermatol..

[10] Hecker A., Schellnegger M., Hofmann E., Luze H., Nischwitz S.P., Karmolz L.P., Kotzbeck P. (2021). The impact of resveratrol on skin wound healing, scarring, and aging. Int. Wound J..

[11] Dai J., Mumper R.J. (2010). Plant phenolics: Extraction, analysis and their antioxidant and anticancer properties. Molecules.

[12] Cheraif K., Bakchiche B., Gherib A., Bardaweel S.K., Ayvaz M.Ç., Flamini G., Ascrizzi R., Ghareeb M.A. (2020). Chemical composition, antioxidant, anti-tyrosinase, anti-cholinesterase and cytotoxic activities of essential oils of six Algerian plants. Molecules.

[13] Uchida R., Ishikawa S., Tomoda H. (2014). Inhibition of tyrosinase activity and melanin pigmentation by 2-hydroxytyrosol. Acta Pharm. Sin. B.

[14] Shahbazi Y. (2017). The properties of chitosan and gelatin films incorporated with ethanolic red grape seed extract and *Ziziphora clinopodioides* essential oil as biodegradable materials for active food packaging. Int. J. Biol. Macromol..

[15] Anbazhagan D., Mansor M., Yan G.O.S., Yusof M.Y.M., Hassan H., Sekaran S.D. (2012). Detection of quorum sensing signal molecules and identification of an autoinducer synthase gene among biofilm forming clinical isolates of *Acinetobacter* spp.. PLoS ONE.

[16] González R.H., Nusblat A., Nudel B.C. (2001). Detection and characterization of quorum sensing signal molecules in *Acinetobacter* strains. Microbiol. Res..

[17] Defoirdt T., Brackman G., Coenye T. (2013). Quorum sensing inhibitors: How strong is the evidence?. Trends Microbiol..

[18] Belizón M., Fernández-Ponce M.T., Casas L., Mantell C., Martínez De La Ossa-Fernández E.J. (2018). Supercritical impregnation of antioxidant mango polyphenols into a multilayer PET/PP food-grade film. J. CO_2_ Util..

[19] Singh R.S., Kaur N., Kennedy J.F. (2019). Pullulan production from agro-industrial waste and its applications in food industry: A review. Carbohydr. Polym..

[20] Spence K.L., Venditti R.A., Rojas O.J., Pawlak J.J., Hubbe M.A. (2011). Water vapor barrier properties of coated and filled microfibrillated cellulose composite films. BioResources.

[21] Tong Q., Xiao Q., Lim L.T. (2013). Effects of glycerol, sorbitol, xylitol and fructose plasticisers on mechanical and moisture barrier properties of pullulan-alginate-carboxymethylcellulose blend films. Int. J. Food Sci. Technol..

[22] Xu W., Zhang F., Luo Y., Ma L., Kou X., Huang K. (2009). Antioxidant activity of a water-soluble polysaccharide purified from *Pteridium aquilinum*. Carbohydr. Res..

[23] Arcan I., Yemenicioglu A. (2011). Incorporating phenolic compounds opens a new perspective to use zein films as flexible bioactive packaging materials. Food Res. Int..

[24] Cui H., Surendhiran D., Li C., Lin L. (2020). Biodegradable zein active film containing chitosan nanoparticle encapsulated with pomegranate peel extract for food packaging. Food Packag. Shelf Life.

[25] Parekh J., Jadeja D., Chanda S. (2005). Efficacy of aqueous and methanol extracts of some medicinal plants for potential antibacterial activity. Turk. J. Biol..

[26] Shan B., Cai Y.Z., Brooks J.D., Corke H. (2007). The in vitro antibacterial activity of dietary spice and medicinal herb extracts. Int. J. Food Microbiol..

[27] Muhs A., Lyles J.T., Parlet C.P., Nelson K., Kavanaugh J.S., Quave C.L. (2017). Virulence inhibitors from Brazilian peppertree block quorum sensing and abate dermonecrosis in skin infection models. Sci. Rep..

[28] Huang Y.L., Chen C.C., Chen Y. (2001). Identification and quantification of major polyphenols in grape seed. J. Nat. Prod..

[29] Luís Â., Neiva D., Pereira H., Gominho J., Domingues F., Duarte A.P. (2014). Stumps of *Eucalyptus globulus* as a source of antioxidant and antimicrobial polyphenols. Molecules.

[30] Gonçalves J., Ramos R., Luís Â., Rocha S., Rosado T., Gallardo E., Duarte A.P. (2019). Assessment of the bioaccessibility and bioavailability of the phenolic compounds of *Prunus avium* L. by in vitro digestion and cell model. ACS Omega.

[31] Scherer R., Godoy H.T. (2009). Antioxidant activity index (AAI) by the 2,2-diphenyl-1-picrylhydrazyl method. Food Chem..

[32] Luís Â., Duarte A., Gominho J., Domingues F., Duarte A.P. (2016). Chemical composition, antioxidant, antibacterial and anti-quorum sensing activities of *Eucalyptus globulus* and *Eucalyptus radiata* essential oils. Ind. Crops Prod..

[33] Sigma-Aldrich Tyrosinase Assay Kit. https://www.sigmaaldrich.com/PT/en/product/sigma/mak550.

[34] Sigma-Aldrich Neutrophil Elastase Activity Assay Kit (Fluorometric). https://www.sigmaaldrich.com/PT/en/product/sigma/mak246.

[35] Ramos A., Rodilla J.M., Ferreira R., Luís Â. (2025). Enhancing hydrophobicity of nanocellulose-based films by coating with natural wax from *Halimium viscosum*. Appl. Sci..

[36] (1995). Paper and Board—Determination of Grammage.

[37] (2011). Paper and Board—Determination of Thickness, Density and Specific Volume.

[38] Luís Â., Domingues F., Ramos A. (2019). Production of hydrophobic zein-based films bioinspired by the lotus leaf surface: Characterization and bioactive properties. Microorganisms.

[39] (2013). Paper—Determination of Transmittance by Diffuse Reflectance Measurement.

[40] (2008). Paper and Board—Determination of Tensile Properties—Part 2: Constant Rate of Elongation Method (20 mm/min).

[41] Luís Â., Pereira L., Domingues F., Ramos A. (2019). Development of a carboxymethyl xylan film containing licorice essential oil with antioxidant properties to inhibit the growth of foodborne pathogens. LWT-Food Sci. Technol..

[42] (2022). Standard Test Methods for Gravimetric Determination of Water Vapor Transmission Rate of Materials.

[43] Luís Â., Ramos A., Domingues F. (2020). Pullulan films containing rockrose essential oil for potential food packaging applications. Antibiotics.

[44] Luís Â., Ramos A., Domingues F. (2021). Pullulan–apple fiber biocomposite films: Optical, mechanical, barrier, antioxidant and antibacterial properties. Polymers.

[45] Bilohan M., Ramos A., Domingues F., Luís Â. (2022). Production and characterization of pullulan/paper/zein laminates as active food packaging materials. J. Food Process Preserv..

[46] Nafchi A.M., Tabatabaei R.H., Pashania B., Rajabi H.Z., Karim A.A. (2013). Effects of ascorbic acid and sugars on solubility, thermal, and mechanical properties of egg white protein gels. Int. J. Biol. Macromol..

